# Andrographolide reduces cognitive impairment in young and mature AβPPswe/PS-1 mice

**DOI:** 10.1186/1750-1326-9-61

**Published:** 2014-12-18

**Authors:** Felipe G Serrano, Cheril Tapia-Rojas, Francisco J Carvajal, Juan Hancke, Waldo Cerpa, Nibaldo C Inestrosa

**Affiliations:** Centro de Envejecimiento y Regeneración (CARE), Santiago, Chile; Departamento de Biología Celular y Molecular, Facultad de Ciencias Biológicas, Pontificia Universidad Católica de Chile, Santiago, Chile; Instituto de Farmacología y Morfofisiología, Universidad Austral de Chile, Valdivia, Chile; Center of Healthy Brain Aging, School of Psychiatry, Faculty of Medicine, University of New South Wales, Sydney, Australia; Centro UC Síndrome de Down, Pontificia Universidad Católica de Chile, Santiago, Chile; Centro de Excelencia en Biomedicina de Magallanes (CEBIMA), Universidad de Magallanes, Punta Arenas, Chile; CARE Biomedical Center, P. Catholic University of Chile, Postal code 8331150 PO Box 114-D, Santiago, Chile

**Keywords:** Alzheimer’s disease, AβPP/PS-1 mice, Water maze, LTD, GSK-3β

## Abstract

Alzheimer’s disease (AD) is a neurodegenerative disorder in which the amyloid-β (Aβ) oligomers are a key factor in synaptic impairment and in spatial memory decline associated with neuronal dysfunction. This impairment includes synaptic failure associated with the loss of synaptic proteins that contribute to AD progression. Interestingly, the use of natural compounds is an emergent conceptual strategy in the search for drugs with therapeutic potentials for treating neurodegenerative disorders. In the present study, we report that andrographolide (ANDRO), which is a labdane diterpene extracted from *Andrographis paniculata*, increases slope of field excitatory postsynaptic potentials (fEPSP) in the CA1 region of hippocampal slices and inhibits long-term depression (LTD), protecting the long-term potentiation (LTP) against the damage induced by Aβ oligomers *in vitro*, most likely by inhibiting glycogen synthase kinase-3β (GSK-3β). Additionally, ANDRO prevents changes in neuropathology in two different age groups (7- and 12-month-old mice) of an AβPPswe/PS-1 Alzheimer’s model. ANDRO reduces the Aβ levels, changing the ontogeny of amyloid plaques in hippocampi and cortices in 7-month-old mice, and reduces *tau* phosphorylation around the Aβ oligomeric species in both age groups. Additionally, we observed that ANDRO recovers spatial memory functions that correlate with protecting synaptic plasticity and synaptic proteins in two different age groups. Our results suggest that ANDRO could be used in a potential preventive therapy during AD progression.

**Electronic supplementary material:**

The online version of this article (doi:10.1186/1750-1326-9-61) contains supplementary material, which is available to authorized users.

## Introduction

Alzheimer’s disease (AD), which is the most common cause of dementia, currently has no known cure. The underlying neuropathology of AD includes extracellular deposition of amyloid β-peptide (Aβ) and intra-neuronal accumulation of hyperphosphorylated *tau* forms [1, 2], as well as synapse dysfunction and neuronal loss [2–5]. An analysis of AD patients’ brains supports the hypothesis that Aβ aggregates are responsible for synapxztic failure [6], and the generation of animal models that reproduce the characteristic features of AD have great relevance to improving the understanding of this disease and to developing new therapies [7, 8].

*Andrographis paniculata* is a native plant from Southeast Asian countries. For centuries, this plant has been used as an official herbal medicine in China for the treatment of various human illnesses, including acute hepatitis, meningitis, choriocarcinoma, malaria, and many other acute inflammatory conditions that can be studied using different animal models [9, 10]. Previous studies have indicated that andrographolide (ANDRO), which is a diterpene of the labdane family, is responsible for most of the biological effects of *Andrographis paniculata* (Additional file 1: Figure S1a) [11–13]. Some studies have suggested that ANDRO might exert neuroprotective effects, i.e., against damage induced by dopamine in mesencephalic neuron-glial cultures associated with a protective effect on inflammation-mediated neurodegeneration [14], oxidative stress induced by nicotine in the brain [15], and cerebral ischemia [16] by inhibiting certain pathways related to inflammation and apoptosis, including Akt, NF-κB and MAPK signaling [13, 17, 18]. Additionally, ANDRO is an apolar compound of low molecular weight that acts on the central nervous system (CNS) in doses of 1 mg/kg and that can cross the blood–brain barrier [16]; thus, ANDRO is an efficient molecule with a potential property for various treatments. However, the role of ANDRO in neurodegenerative diseases, such as AD, has not been investigated.

We designed a set of experiments to determine the potential role of ANDRO in synaptic transmission and in memory using an AD transgenic mouse model with AβPP and PS-1 mutant transgenes (AβPP/PS1) [7]. We study the effect of ANDRO in young and mature transgenic mice (7- and 12-month-old mice, respectively) using behavioral, electrophysiological, biochemical and cytochemical analyses. We observed a recovery of memory, synaptic functions, and long-term potentiation (LTP) and a reduction in *tau* phosphorylation in both groups of animals. Interestingly, we detected a reduction in Aβ species and amyloid plaques in the hippocampus in 7-month-old mice. With this approach, *in vitro* assays indicate that ANDRO causes an increase in the slope of field excitatory postsynaptic potential (fEPSP) over time. Additionally, ANDRO has the capacity to induce a protection of LTP and synaptic proteins against the Aβ oligomers. Also, we found that ANDRO has the property to inhibit the long-term depression (LTD) in a concentration-dependent manner, showing an accumulation of β-catenin and a reduction in the active state of glycogen synthase kinase-3 β (GSK-3β), a key enzyme associated with LTD and Wnt signaling [19–21]. Our results suggest that ANDRO might be beneficial for treating AD.

## Results

### ANDRO decreases Aβ depositions in young AβPPswe/PS-1 mice

**Figure 1:**
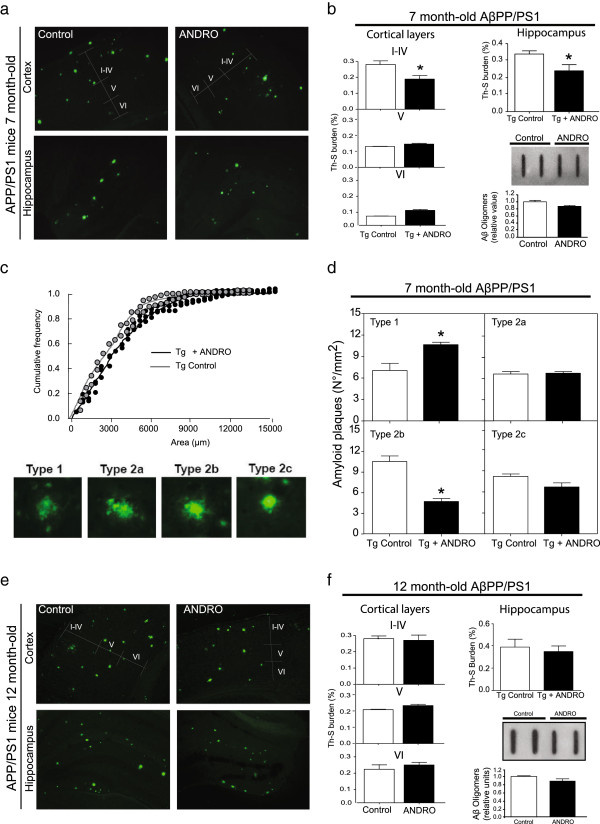
**ANDRO reduces Aβ aggregates in the brains of young AβPPswe/PS-1 mice. (a)** Representative Th-S staining used to detect amyloid deposits in brains of 7-month-old AβPPswe/PS-1 mice with vehicle and ANDRO treatment. **(b)** The amyloid burden was quantified using Th-S staining and the number per area in different separated cortex layers and in hippocampi. White bars show double AβPPswe/PS-1 control mice, and black bars show AβPPswe/PS-1 ANDRO, at 10x magnification. The right-inferior panel represents the slot blot from hippocampus samples, using 6 μg of protein per slot incubated with the A11 antibody. Normalized densitometry analysis. **(c)** Cumulative frequency plot of amyloid plaque size for control and ANDRO-treated AβPPswe/PS-1 mice. **(d)** Characterization of the amyloid Th-S-positive plaques presented in the brains of 7-month-old AβPPswe/PS-1 mice; type 1, plaques displaying a reticular appearance without a central dense core; type 2, plaques displaying a dense core surrounded by either fibrillar material in the shape of a corona (type 2a) or radiating from the center (type 2b) or displaying a dense core with no surrounding material (type 2c). **(e)** Th-S staining used to detect amyloid deposits in brains of 12-month-old AβPPswe/PS-1 transgenic mice treated with vehicle (left panel) and ANDRO (right panel). **(f)** Amyloid burden was quantified using Th-S staining and the number of amyloid plaques per area in separated cortex layers, and in hippocampi. White bars show AβPPswe/PS-1 control mice, and black bars show AβPPswe/PS-1 ANDRO, at 10x magnification. The right-inferior panel represents the slot blot from hippocampus samples of mature AβPPswe/PS-1 control and ANDRO-treated mice. Graph represents the normalized densitometry analysis. Three animals were used per experimental group. Data are presented as mean ± SEM. Statistical differences were calculated by Student’s t test, followed by Dunnett’s post hoc test. Asterisks indicate statistically significant differences (*p < 0.05).

Previous studies have suggested that amyloid levels in AD patients and mouse models are related to cognitive impairment [22]; the effects of Aβ oligomers are thought to be the cause of synaptic function impairment in the postsynaptic region [23–25]. Under this condition, young (7-month-old) and mature (12-month-old) AβPPswe/PS-1 mice were treated with ANDRO for 4 weeks, and then the amounts of Aβ aggregates present in their brains were analyzed. A more detailed analysis of amyloid plaques in the cortical layers and hippocampi were measured in 7-month-old AβPPswe/PS-1 mice (Figure 1a and b). These data show a significant reduction in amyloid plaques in cortical layers I-IV [F(7,3) = 2,68, p < 0,05], whereas we did not observe changes in layers V [F(15,3) = 3,15, p = NS] and VI [F(17,3) = 2,38, p = NS] with ANDRO treatment (Figure 1b) [F(4,4) = 0,1077, p = NS]. Additionally, we detected a reduction in the ThS burden in the hippocampus (Figure 1b) [F(12,3) = 3,15, p < 0,05]. Then, to analyze a probable change in the levels of Aβ oligomers in the hippocampi of 7-month-old AβPPswe/PS-1 mice treated with ANDRO, we evaluated the levels of Aβ oligomers in hippocampi by slot blot using the A11 antibody without finding any significant changes (Figure 1b). These results indicate that ANDRO treatment causes a reduction in the overall amount of Aβ plaques, but not Aβ oligomers, in young animals. In this context, we performed a detailed analysis of the plaque size distribution. The aggregate size distribution, which is presented as a cumulative frequency plot, demonstrates that AβPPswe/PS-1 mice treated with ANDRO shifted their plaque size distribution toward a smaller plaque size from cortex (Figure 1c). To further analyze the effect of ANDRO treatment on amyloid plaques, we performed an analysis of the different amyloid plaques, which were categorized by maturation stages according to their morphology as follows [26]: type 1, plaques displaying a reticular appearance without a central dense core; type 2, plaques displaying a dense core surrounded by fibrillar material in the shape of a corona (type 2a) or radiating from the center (type 2b) or plaques displaying a dense core lacking any surrounding material (type 2c) (Figure 1d). The analysis of type 2a [F(1,3) = 1,5, p = NS]and 2c[F(1,3) = 3,1, p = NS]plaques in the whole brains of 7-month-old AβPPswe/PS-1 mice treated with ANDRO indicated no significant differences in relation to controls. In contrast, the number of type 2b plaques significantly decreased in the brains of ANDRO-treated AβPPswe/PS-1 young mice (Figure 1d) [F(2,3) = 5,13, p < 0,05]. The number of immature plaques (type 1) increased significantly with ANDRO treatment, demonstrating that ANDRO treatment affects amyloid plaque maturation [F(2,3) = 13,54, p < 0,05].

In contrast, the analysis of Aβ-aggregates in mature AβPPswe/PS-1 mice indicated that 12-month-old AβPPswe/PS-1 Control mice showed high levels of amyloid plaques in the hippocampus and in the cortex (Figure 1e); the quantification of the amyloid burden area is shown in Figure 1f. ANDRO was not able to affect amyloid deposits levels in the hippocampus [F(2,3) = 20.08, p = NS] or in the cortex (layer I-IV [F(17,3) = 2,38, p = NS], V [F(16,3) = 21.3, p = NS], VI [F(18,3) = 19.71, p = NS], and no significant changes in the levels of Aβ oligomers were observed (Figure 1f) [F(4,4) = 0,0941, p = NS]. These results indicate that ANDRO does not affect the Aβ load in the mature AβPPswe/PS-1 mice; however, a significant decrease in Aβ aggregates was observed in young transgenic mice, suggesting that ANDRO prevents Aβ aggregation in early stages during disease development in this animal model.

### *Tau*phosphorylation decreases after ANDRO treatment in both young and mature AβPPswe/PS-1 mice

**Figure 2:**
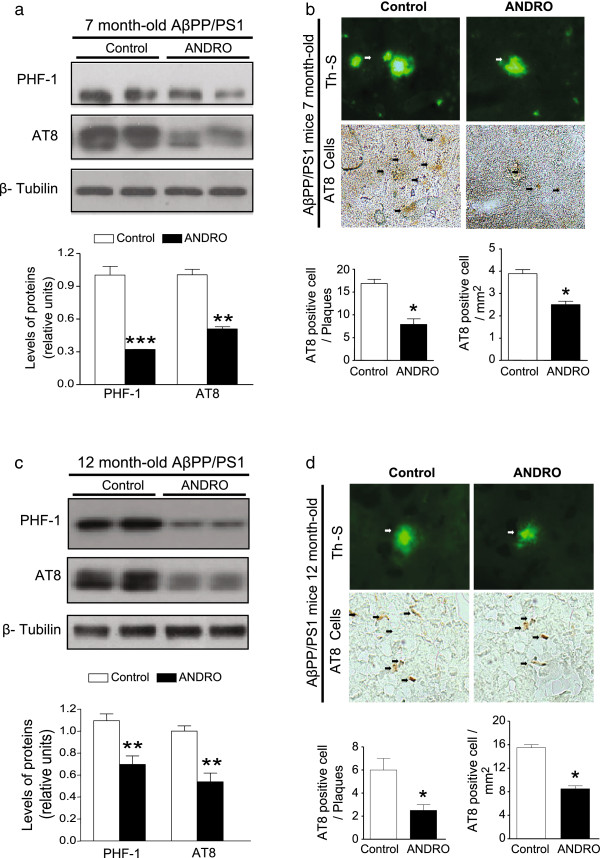
**ANDRO reduces tau phosphorylation in the brains of AβPPswe/PS1 mice of different ages. (a)** Immunoblot of hippocampus homogenates from young (7-month-old) AβPPswe/PS-1 mice treated with vehicle (control, white bars) or ANDRO (black bars) using the PHF-1 and AT8 antibodies. Graph corresponds to the densitometric analysis of bands normalized against β-tubulin (loading control) and compared with AβPPswe/PS-1 mice treated with vehicle or with ANDRO. **(b)** AT8-positive cells are detected in other regions in young (7-month-old) AβPPswe/PS-1 control or ANDRO-treated mice. Graphs show the quantification of the number of AT8-positive neurons per area in mm^2^ and near amyloid plaques, as detected by ThS staining. Positive neurons and amyloid plaques are indicated by black and white arrows, respectively. **(c)** Immunoblot of hippocampus homogenates from mature (12-month-old) AβPPswe/PS-1 control and ANDRO-treated mice using the PHF-1 and AT8 antibodies. Graph corresponds to the densitometric analysis of bands normalized against a loading control and compared with AβPPswe/PS-1 control and ANDRO-treated mice. **(d)** AT8-positive cells are detected in other regions in AβPPswe/PS-1 control and ANDRO-treated mice. The graphs show the quantification of the number of AT8-positive neurons per area in mm^2^ and near amyloid plaques, as detected by ThS staining. Positive neurons and amyloid plaques are indicated by black and white arrows, respectively. Analysis of AT8-positive neurons per plaque. Three animals were used per experimental group. Data are presented as mean ± SEM. Statistical differences were calculated by Student’s t test, followed by Dunnett’s post hoc test. Asterisks indicate statistically significant differences (*p < 0.05).

Another critical AD hallmark is *tau* pathology [1], which is characterized by the hyperphosphorylation of the *tau* protein, which generates PHF accumulation and neurofibrillary tangle formation [1]. In our model, *tau* protein phosphorylation in AD epitopes has been described in mature animals [27, 28], and Aβ induces the manifestation of phospho-epitopes in *tau* proteins, which is associated with neuronal damage [29]. Using immunoblotting, we measured *tau* phosphorylation residues associated with AD that correspond to Ser-396-Ser-404 (PHF-1) and the Ser-202 (AT8) residue (Figure 2) [30]. We found a reduction in PHF-1- and AT8-positive *tau* levels in the hippocampi of 7-month-old AβPPswe/PS-1 mice treated with ANDRO in comparison with AβPPswe/PS-1 untreated mice (Figure 2a) [PHF1: F(6,4) = 0.0003, p < 0.05, AT8: [F(6,4) = 5.084, p < 0,05]]. Consistent with this idea, previous reports have indicated that increased *tau* phosphorylation in specific phosphorylation epitopes surrounding the plaque is a sign of Aβ-induced neuronal damage [1]. Accordingly, we studied the appearance of the AT8 *tau* epitope near Aβ plaques (Figure 2b). Although *tau* phosphorylation affects different parts of the hippocampus and cortex, we chose to evaluate AT8-positive cells in a circular area (r ≈ 100 mm) surrounding amyloid plaques [31], where cytoskeletal changes, *tau* phosphorylation, GFAP protein activation and synapse loss have been found to occur [2, 31]. ANDRO treatment induced a clear decrease in the number of AT8-positive neurons next to amyloid deposits for 7-month-old AβPPswe/PS-1 mice (Figure 2b) [F(17,3) = 4,7, p < 0.05]. The analysis of the total number of AT8-positive cells found outside the circular area around the plaques in the hippocampus indicated a decrease in the number of these cells after ANDRO treatment (Figure 2b) [F(2,3) = 1,4, p < 0.05]. Additionally, we analyzed the effect of ANDRO in 12-month-old AβPPswe/PS-1 mice and observed a significant reduction in the PHF-1- and AT8-positive *tau* levels in the hippocampus (Figure 2c) [PHF1: F(6,4) = 3,1, p < 0,05, AT8: F(6,4) = 4.7, p < 0,05]. Interestingly, the analysis of the total number of AT8-positive cells [F(2,3) = 4.0, p < 0.05], and AT8 positive cells found outside the circular area around the plaques in the hippocampus indicated a decrease in the number of these cells after ANDRO treatment (Figure 2d) [F(1,3) = 16, p < 0.05]. These results clearly indicate that ANDRO is able to prevent and reverse *tau* phosphorylation, which is a key event in AD pathology in young and mature AβPPswe/PS-1 mice.

### ANDRO recovers the synaptic functions in AβPPswe/PS1 mice of different ages

**Figure 3:**
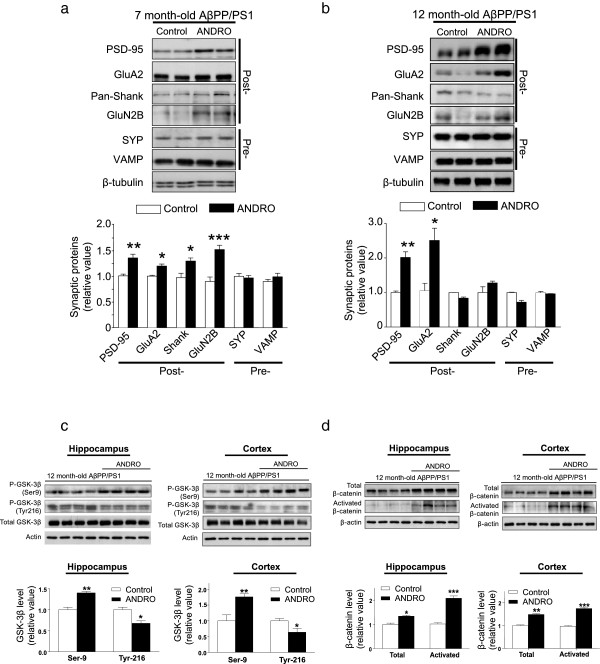
**ANDRO recovers synaptic proteins, increase levels of β-catenin and reduce levels of active of GSK-3β. (a)** Immunoblots of total postsynaptic proteins (PSD-95, GluA2, Shank and GluN2B) and presynaptic proteins (SYP and VAMP) extracts from the hippocampi of 7-month-old AβPP**/**PS1 control and ANDRO-treated mice (white and black bars, respectively). The graph corresponds to the densitometric analysis of each postsynaptic and presynaptic proteins normalized against β-tubulin and compared with the levels of the same protein in AβPP/PS1 control mice. Immunoblots of total protein extracts from the hippocampi of 12-month-old AβPP**/**PS1 control and ANDRO-treated mice (white and black bars, respectively). **(b)** Immunoblots of total postsynaptic proteins (PSD-95, GluA2, Shank and GluN2B) and presynaptic proteins (SYP and VAMP) extracts from the hippocampi of 12-month-old AβPP**/**PS1 control and ANDRO-treated mice (white and black bars, respectively). Graph corresponds to the densitometric analysis of each postsynaptic and presynaptic proteins normalized against β-tubulin and compared with the levels of the same protein in AβPP/PS1 control mice, n ≥ 3. *p < 0.05; **p < 0.01; ***p < 0.001. **(c)** Immunoblots of total GSK-3β, inactive form of GSK-3β (pGSK3β^ser9^) and active form of GSK-3β (pGSK3β^tyr216^) proteins extracts from the hippocampi and cortex of 12-month-old AβPP**/**PS1 control and ANDRO-treated mice (white and black bars, respectively). **(d)** Immunoblots of total β-catenin and activated form of β-catenin proteins extracts from the hippocampi and cortex of 12-month-old AβPP**/**PS1 control and ANDRO-treated mice (white and black bars, respectively) Graph corresponds to the densitometric analysis of each postsynaptic and presynaptic proteins normalized against actin and compared with the levels of the same protein in AβPP/PS1 control mice, n ≥ 3. *p < 0.05; **p < 0.01; ***p < 0.001.

According to the present evidence, the proteins that determine the structure and function of the synapses are diminished in AD brains and are altered in transgenic AD models [3–5, 32]. Therefore, we performed an analysis of the affected synaptic proteins in the hippocampi and cortices of AβPPswe/PS-1 mice by immunoblotting. We evaluated the levels of the synaptic vesicle protein synaptophysin (SYP) and the synaptic vesicle-associated integral membrane protein (VAMP), which are pre-synaptic markers (Pre-). Additionally, we evaluated the levels of the synaptic scaffold protein Shank, the NMDA receptor subunit GluN2B, the AMPA receptor subunit GluA2 and the postsynaptic-density-protein 95 (PSD-95) [33], which are postsynaptic markers (Post-). As expected, ANDRO treatment significantly recovered the post-synaptic proteins in the hippocampi of young AβPPswe/PS-1 mice (Figure 3a) [PSD-95: F(6,4) = 4.6, p < 0.05, GluA2: F(6,4) = 2.0, p < 0.05, Shank: F(6,4) = 3.2, p < 0.05, GluN2B: F(6,4) = 6.1, p < 0.05], whereas the total levels of pre-synaptic proteins SYP and VAMP in the hippocampi of young AβPPswe/PS-1 mice were unaltered (Figure 3a) [SYP: F(6,4) = 0.4, p = NS, VAMP: F(6,4) = 2.0, p = NS]. We also quantified the levels of synaptic proteins in 12-month-old AβPPswe/PS-1 mice treated with ANDRO. We observed an increase in PSD-95 and GluA2 levels (Figure 3b) [PSD-95: F(6,4) = 5.9, p < 0.05, GluA2: F(6,4) = 3.4, p < 0.05, Shank: F(6,4) = 1.0, p = NS, GluN2B: F(6,4) = 1.2, p = NS] and the total levels of pre-synaptic proteins SYP and VAMP in the hippocampi of old AβPPswe/PS-1 mice were unaltered (Figure 3b) [SYP: F(6,4) = 1.1, p = NS, VAMP: F(6,4) = 0.6, p = NS], indicating that inducing a recovery of certain proteins associated with the postsynaptic structure and with transmission remains possible in advanced neurodegeneration models [33]. This evidence suggests that ANDRO prevents the loss of postsynaptic proteins in 7-month-old mice and allows the recovery of synaptic proteins in mature transgenic animals.

In this context, we has reported previously that in the transgenic model exist an increase in the activity of GSK-3β [32, 34, 35]. Therefore, we evaluate the levels of the active form of GSK-3β in cortex and hippocampus of 12 month-old AβPPswe/PS-1 mice treated with ANDRO (Figure 3c). We observed that ANDRO increase the levels of the inactive form of GSK-3β (phosphorylation in Ser-9) while the levels of active form (phosphorylation in Tyr-216) of this enzyme remains reduced (Figure 3c). This evidence suggests that ANDRO plays a role in the protection of proteins related to synaptic processes and enzymes related with the neuropathology of AD.

### ANDRO recovers the spatial memory in AβPPswe/PS-1 double transgenic mice of different ages

**Figure 4:**
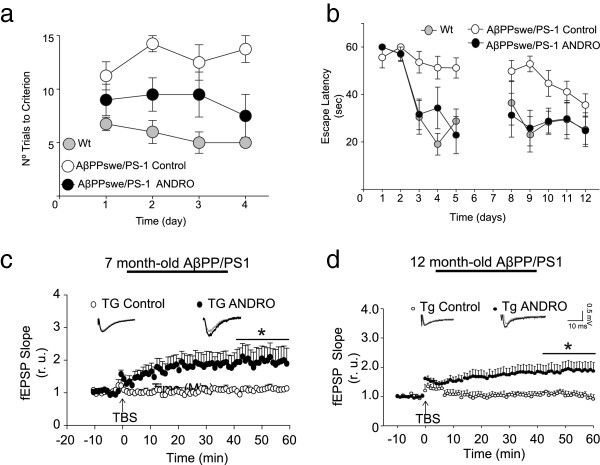
**ANDRO recovers the cognitive functions and LTP in an AD mouse model of different ages** ***.*** **(a)** Behavioral performance in the memory flexibility test. Seven-month-old AβPPswe/PS-1 mice treated with control vehicle solution (Wt, gray circles and AβPPswe/PS-1 control, white circles) or with ANDRO (AβPPswe/PS-1 ANDRO, black circles). **(b)** Behavioral performance was evaluated by the escape latency in the classical MWM test. Twelve-month-old Wt and AβPPswe/PS-1 mice were treated with vehicle solution (Wt, gray circles and AβPPswe/PS-1 control, white circles) or with ANDRO (AβPPswe/PS-1 ANDRO, black circles), n ≥ 5. *p < 0.05. **(c)** LTP was generated by TBS in hippocampal CA1 in AβPP/PS1 slices from 7-month-old ANDRO-treated mice (black circle) shows a recovery in the capacity to induce LTP in comparison with AβPP/PS1 control mice (white circles). **(d)** LTP generated by TBS in hippocampal CA1 in AβPP/PS1 slices from 12-month-old mice treated with vehicle solution or with ANDRO shows a recovery in the capacity to induce LTP in comparison with AβPP/PS1 control mice (white circles). The dots and bars represent the mean ± SE from 7 different slices. *p < 0.05. Data are presented as means ± S.E.M. Three animals were used per experimental group. Data are presented as mean ± SEM. Statistical differences were calculated by Student’s t test, followed by Dunnett’s post hoc test. Asterisks indicate statistically significant differences (*p < 0.05). Statistical significant differences in behavioral experiments were calculated by one-way ANOVA, followed by Bonferroni’s post hoc test.

We evaluated the hippocampal function associated with spatial memory using a behavioral task related to learning and memory in young and mature AβPPswe/PS-1 mice treated with ANDRO (Figure 4) [36]. Previous evidence has indicated that the cognitive deficit associated with spatial memory in young AβPPswe/PS-1 mice is highly variable [32]. Therefore, we evaluated the performance of 7-month-old AβPPswe/PS-1 mice using a modified spatial memory paradigm associated with episodic memory (memory flexibility) that has been proven to be more sensitive for detecting hippocampal dysfunctions [37]. The analysis of the behavioral performance indicated that ANDRO-treated AβPPswe/PS-1 mice required fewer trials to achieve the learning criterion in comparison with the AβPPswe/PS-1 control mice (Figure 4a) [ANOVA p < 0.05, F(2,6) = 19.00]. Additionally, wild-type animals required fewer trials to achieve the criterion (Figure 4). We evaluated the spatial memory performance in 12-month-old AβPPswe/PS-1 mice using the Morris water maze (MWM). The results indicated that these mice had the highest latency values, which is consistent with hippocampal dysfunction triggered by Aβ neurotoxic effects [32]. In contrast, wild-type animals injected with vehicle had normal escape latency values during training. However, ANDRO-treated AβPPswe/PS-1 mice presented significantly lower escape latency values, similar to wild-type mice (Figure 4b), indicating that ANDRO was able to reduce the cognitive impairment in the spatial memory performance during the first and the second weeks of training. These results indicated that ANDRO has beneficial effects on the cognitive impairment present in young and mature AβPPswe/PS-1 transgenic mice.

Consistent with the idea of a reduction of certain AD hallmarks and a recovery of synaptic proteins and cognitive functions, we evaluated the synaptic plasticity of the two groups of AβPPswe/PS-1 mice (7- and 12-month-old) by studying the LTP magnitude in the hippocampal CA3-CA1 transmission, which also correlates with memory and learning processes [25, 36, 38]. We did not observe LTP induction in untreated AβPPswe/PS-1 mice from different age, which is consistent with previous studies [38] (Figure 4c and d). Nevertheless, 7-month-old AβPPswe/PS-1 mice treated with ANDRO had the ability to induce LTP, which was maintained for at least 1 h (Figure 4c) [F(8,5) = 17.66, p < 0,05]. Additionally, no LTP was observed in the untreated 12-month-old AβPPswe/PS-1 mouse model (Figure 4d) [F(4,5) = 5.964, p < 0,05]. However, LTP induction reappears after ANDRO treatment in comparison with 12-month-old AβPPswe/PS-1 animals (Figure 4d). Additionally, we did not observe a change in LTP induction in ANDRO-treated wild-type animals in either age group (Additional file 2: Figure S2a and b). These results suggest that ANDRO protects the synaptic architecture and function in transgenic AD mice, allowing the induction of synaptic processes. In wild-type animals, the synaptic structure and function are intact, and no protection by ANDRO is required.

### ANDRO inhibits GSK-3β, preventing LTD induction

**Figure 5:**
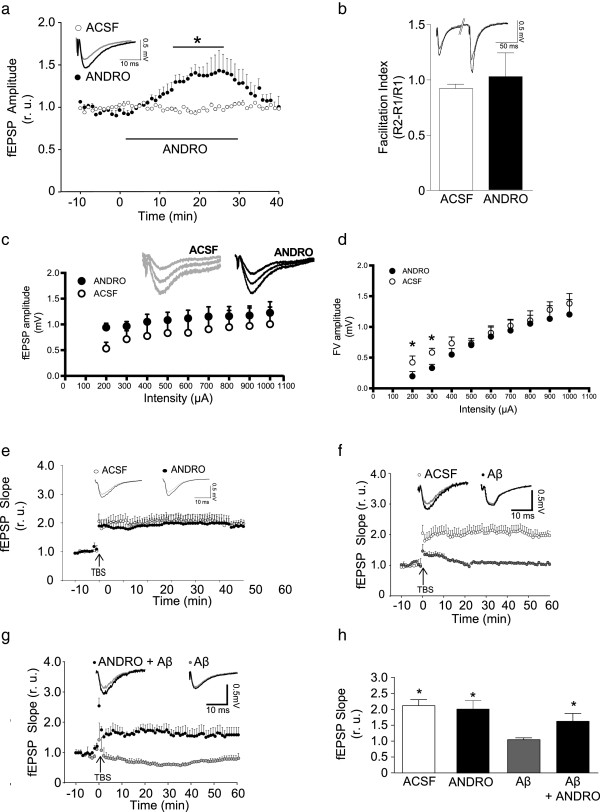
**ANDRO increases the synaptic transmission and protect the LTP against the Aβ oligomers in vitro. (a)** fEPSP recordings in the presence of 10 μM ANDRO. **(b)** Plot of paired pulse facilitation (PPF) in the presence or absence of ANDRO. Inset shows representative recordings. Bars represent the mean ± SE from 7 different slices, *p < 0.05. **(c)** fEPSP amplitude induced by the input–output protocol treated with ANDRO (black circle) or with vehicle solution (ACSF, white circles). **(d)** Fiber volley (FV) amplitude induced by the input–output protocol treated with ANDRO (black circle) or with vehicle solution (white circles). **(e)** Hippocampal slices were exposed to ANDRO, and LTP was induced. The arrow indicates LTP induction by TBS, and the plot shows the fEPSP slope at different times. **(f)** Hippocampal slices were exposed to Aβ oligomers (1 μM); arrow indicates the time of TBS and the plot show the fEPSP slope at different times. **(g)** Hippocampal slices were exposed to ANDRO (10 μM) in the presences of Aβ oligomers (1 μM), arrow indicates the time of TBS and the plot show the fEPSP slope at different times. **(h)** Plot of fEPSP slope changes, in the presence or absence of ANDRO plus Aβ oligomers. The inset shows representative recordings. The dots and bars represent the mean ± SE from 7 different slices, *p < 0.05. Three animals were used per experimental group. Data are presented as mean ± SEM. Statistical differences were calculated by Student’s t test, followed by Dunnett’s post hoc test. Asterisks indicate statistically significant differences (*p < 0.05). Statistical significant differences in in vitro experiments of ANDRO and Aβ oligomers were calculated by one-way ANOVA, followed by Bonferroni’s post hoc test.

We decided to evaluate the role of ANDRO in synaptic transmission *in vitro*. For this evaluation, we incubated hippocampal slices from WT mice from 2 month with 10 μM ANDRO for 30 min and observed an increase in the slope of fEPSP (Figure 5a) [F(3,5) = 49.39, p < 0,05]. To investigate whether this effect corresponded to a pre- or postsynaptic effect, the PPF index was determined [39]. The results indicated that ANDRO did not change the facilitation index (Figure 5b) [F(4,4) = 35.15, p = NS], indicating that the response did not depend on presynaptic modulation and is most likely mediated by a postsynaptic effect. Interestingly, we performed input–output experiments to analyze synaptic strength; however, we did not observe any effects on the basal transmission when the samples were exposed to ANDRO (Figure 5c and d).

Additionally, we decided to evaluate the contribution of ANDRO in synaptic plasticity, studying the modulation of LTP and LTD, which correlate with memory and learning processes [40]. We observed that incubation for 1 h with ANDRO did not induce a change in the LTP formation (Figure 5e); however, interestingly, incubations of Aβ oligomers, which are reported to inhibit the induction of LTP [6, 41] (Figure 5f) in presence of ANDRO induced LTP, indicating a protection of synaptic plasticity in the slices (Figure 5g and h) [ANOVA p < 0.05, F(3,14) = 5.236]. In order to obtain more evidence for the neuroprotective effect of ANDRO on the damage induced for Aβ, we explored hippocampal slices exposed to Aβ oligomers for 1 h. These slices showed reduced levels of the postsynaptic proteins GluN2B, GluA2 and PSD-95 as compared to hippocampal slices treated with ACSF and we do not see differences in presynaptic components (Additional file 3: Figure S3a, [GluN2B: ANOVA p < 0.05, F(3,8) = 28,28, GluA2: ANOVA p < 0.05, F(3,8) = 7,656, PSD-95: ANOVA p < 0.05, F(3,8) = 12,07, SYP: ANOVA p = NS, F(3,8) = 1,807 and VAMP: ANOVA p < 0.05, F(3,8) = 5,239). Therefore, we found a critical role of ANDRO in LTP and synaptic protein neuroprotection in the presence of Aβ oligomers *in vitro*, suggesting that ANDRO could recover the neuronal functions without alter the levels of amyloid in our AD model.

**Figure 6:**
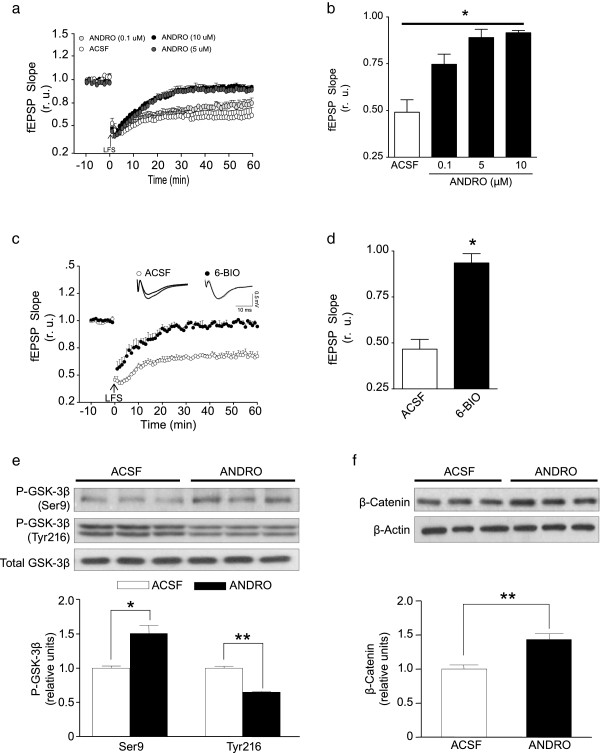
**ANDRO inhibits GSK-3β, increases β-catenin levels and inhibits LTD induction. (a)** Hippocampal slices from wild-type animals were exposed to different concentrations of ANDRO (0.1, 5 and 10 μM, black circles) to inhibit the LTD in the CA1 hippocampal region. The arrow indicates the time of LFS, and the plot shows the fEPSP slope at different times. **(b)** The right graph represents the quantification of the LTD magnitude to 60 min post-induction in the presence of ANDRO (black bars). **(c)** Hippocampal slices from wild-type mice were exposed to 6-BIO (10 nM), and LTD was induced. The arrow indicates the time of LFS, and the plot shows the fEPSP slope at different times. The inset shows representative recordings. **(d)** Plot of changes in fEPSP slope in the presence or absence of 6-BIO. **(e)** Quantification shows that the presence of ANDRO (black bars) induces an increase in the phosphorylation of GSK-3β (pGSK3βser9) and a reduction in pGSK3βtyr216 compared with ACSF treatment (white bars). Each protein was normalized against total GSK-3β. **(f)** β-catenin was evaluated in the presence of ANDRO (black bars) or ACSF (white bars). Densitometric analysis of each protein normalized against total β-catenin and β-actin (loading control) were compared with the levels of the same protein in hippocampal slices from wild-type animals. Bars represent the mean ± SE n ≥ 3. *p < 0.05; **p < 0.01. The inset shows representative recordings. The dots are the mean ± SE from 7 different slices, n ≥ 3. * p < 0.05. Three animals were used per experimental group. Data are presented as mean ± SEM. Statistical differences were calculated by Student’s t test, followed by Dunnett’s post hoc test.. Statistical significant differences in dosis response were calculated by one-way ANOVA, followed by Bonferroni’s post hoc test.

Interestingly, another process of long-term plasticity is LTD, which participate in decrease the synaptic strength and is related with the signaling of Aβ peptide [42]. We found that the LTD induction was inhibited by ANDRO in a concentration-dependent manner (0.1 μM, 5 μM and 10 μM, Figure 6a and b) [ANOVA p < 0.05, F(3,14) = 6.630]. To evaluate a modulator of LTD, we decided to analyze GSK-3β, which is a key enzyme studied in neurodegenerative diseases and in the processes of plasticity and memory [20, 43]. In particular, GSK-3β plays a role in the internalization of AMPA receptors from the synaptic spine [20], modulates PSD-95 [43], and controls some events of LTD [19, 20, 44]). The use of 10 nM 6-BIO, which is a selective inhibitor of GSK-3β [45], produced LTD inhibition similar to the effect observed with ANDRO in hippocampal slices (Figure 6c and d) [F(5,5) = 1.113 p < 0.05].

Therefore, we incubated hippocampal slices for 1 h with ANDRO and evaluated the protein composition by immunoblotting (Figures 6e and f). We observed an increase in the inactive state of GSK-3β, which is associated with serine 9 (Ser9) phosphorylation on GSK-3β (Figure 6e). Additionally, the active state, which is identified by tyrosine 216, was reduced (Figure 6e) [Ser9, F(4,4) = 4.1, p < 0.05 and Tyr216: F(4,4) = 6.0, p < 0.05]. In complementary experiments, we evaluated β-catenin, which is a protein downstream of GSK-3β that is phosphorylated by GSK-3β, determining its degradation [46]. We observed an increase in the β-catenin levels in slices incubated with ANDRO, suggesting a decrease in GSK-3β activity (Figure 6f) [β-catenin, F(4,4) = 5.9, p < 0.05]. Additionally, reports indicate that the presence of Aβ induces an activation of the GSK-3β and a decrease of β-catenin [34, 47] (Additional file 3: Figure S3b, Ser9: F(4,4) = 3.6, p < 0.05, Tyr216: F(4,4) = 4.2, p < 0.05 and β-catenin: F(4,4) = 6.4, p < 0.05). However, the presence of ANDRO reduce the active form of GSK-3β and recover the levels of β-catenin (Additional 3: Figure S3c, Ser9: F(4,4) = 6.2, p < 0.05, Tyr216: F(4,4) = 4.0, p < 0.05 and β-catenin: F(4,4) = 4.8, p < 0.05]) replicating with an *in vitro* model the changes induced in the transgenic model (Figure 3c) [34]. This evidence suggests that ANDRO plays a role in the modulation of synaptic plasticity associated with LTD and that this role is most likely related to GSK-3β inhibition.

## Discussion

In the present study, we observed that young (7-month-old) and mature (12-month-old) AβPPswe/PS-1 mice treated with ANDRO recovered cognitive capabilities and showed a decrease in several neuropathological markers of AD. A specific neuroprotective effect of ANDRO was observed in AβPPswe/PS-1 mice in this study, including protection of postsynaptic proteins, reduction of Aβ aggregate maturation, recovery of synaptic functions, such as LTP, and recovery of spatial memory performance. Interestingly, the differences between ages in both AD model present variations in the damage of cognition, implicating that 7-month old mice do not present changes in spatial memory measured by classical Morris water maze test. However, these mice present alterations in their episodic memory evaluated by the memory flexibility test [32, 37, 48]. Additionally, 12 month-old mice that present alterations in spatial memory measured by classical Morris water maze test, present a recovery of their synaptic functions. Interestingly, we observed that the treatment with ANDRO induces a recovery of process of episodic memory in 7 month-old transgenic mice and spatial memory functions in 12 month-old transgenic mice. Moreover, ANDRO inhibits LTD induction in wild-type animals by a mechanism that may include the inhibition of GSK-3β activity. Taken together, our results indicate that ANDRO might be of therapeutic relevance in AD.

Other reports have indicated that the anti-inflammatory effects of ANDRO [12, 13] could be an additional mechanism responsible for alleviating the late pathological alterations observed in the brains of transgenic mice and that these effects might aid in the protective role of ANDRO against the neuroinflammatory response triggered by glial cells [49], limiting the cognitive damage observed in an AD model [50]. Additionally, evidence of the etiopathology of free radicals exists in AD patients [30, 51]. These free radicals trigger post-translational modifications in different proteins, including oxidation, glycation and nitrotyrosination, which are all chemical modifications that stimulate protein function loss. As previously described, all of these effects are particularly relevant in AD [52]. Several studies have reported antioxidant neuroprotective activity against Aβ-mediated cytotoxicity [53, 54]. Therefore, ANDRO may possess some antioxidant properties that play a role in the neuroprotection of the cognitive capacities observed in the present study in the ANDRO-treated AβPPswe/PS-1 transgenic mice.

### Mechanism describing the Aβ oligomer - *tau*interaction and the role of GSK-3β

Recent reports have indicated that the interaction between Aβ oligomers and *tau* proteins could generate damage and increase cell death in the brain [55]. This evidence regarding the amyloid cascade hypothesis suggests that Aβ formation is a critical step in driving AD pathogenesis and that Aβ formation could coexist with the action generated by *tau,* increasing dysregulation signals in AD [2]. In the present study, we demonstrated that *tau* phosphorylation at the PHF-1 and AT-8 sites is reduced, that the cognitive function is recovered and that the loss of LTP induction and maintenance in the CA1 regions of hippocampi is reversed in ANDRO-treated AβPPswe/PS-1 mice. However, we did not observe a decrease in the number or size of amyloid plaques in the hippocampi of mature mice, indicating that the stable plaques formed are not degraded by ANDRO and that ANDRO may play a role in or downstream of the Aβ aggregation pathway. Alternatively, the size or number of senile plaques might not correlate with cognitive impairment [56], and Aβ oligomers might play a more relevant role in synaptotoxicity that alters the cognitive process [23]. Interestingly, we observed a reduction in the amyloid species in 7-month-old transgenic mice, indicating that ANDRO could have therapeutic relevance in stages of plaque formation before the stabilization of senile plaques during the period of advanced age in this AD model.

The use of ANDRO to regulate GSK-3β activity is a new approach compared with other inhibitors. The use of selective inhibitors, such as SB216763 (SB), induces a reduction in the impaired memory generated by intra-cerebroventricular Aβ oligomer infusion [57]. However, the use of SB generates adverse effects in control mouse behavior, indicating that GSK-3β plays an important role in processes such as memory and learning [57]. In contrast, ANDRO treatment does not induce changes in synaptic plasticity processes, such as LTP, in control slices, most likely indicating that no changes occur in the normal LTP process.

These results suggest a functional protection mechanism triggered by the presence of ANDRO, and this protective property might explain its capacity against the cognitive impairment observed in the AβPPswe/PS-1 mice. The ANDRO effect leads to protection of LTP and postsynaptic proteins, and this protection might be another relevant factor in restoring the cognitive impairment at an early stage of the disease. Interestingly, ANDRO treatment at different stages of the AD model protected against synaptic protein loss without altering the Aβ oligomers levels in the transgenic mice. This finding might indicate that the presence of ANDRO could have a potential effect on Aβ oligomer signaling during changes in synaptic transmission and plasticity.

### Role of GSK-3β in synapse impairment by Aβ oligomers

**Figure 7:**
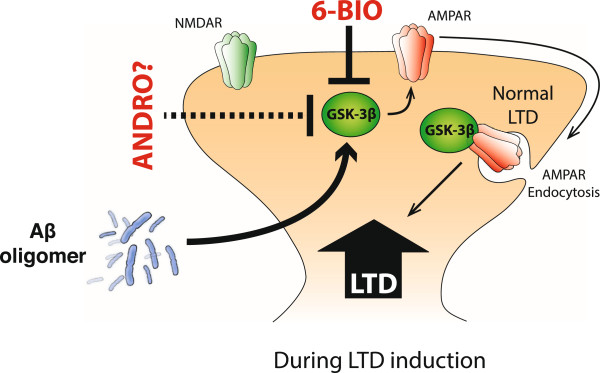
**Mechanism of ANDRO action in the synaptic neuroprotection of Aβ oligomers.** The presence of Aβ oligomers induces an increase in GSK-3β, which facilitates the process of LTD and which contributes to the internalization of AMPA receptors from the postsynaptic region, reducing the synaptic strength. However, the presence of ANDRO induces GSK-3β inhibition, which, in turn, reduces PSD-95 phosphorylation, preventing the internalization of AMPA receptors and inhibiting the Aβ oligomeric effect on synaptic depression.

Additionally, a pathway related to the Aβ oligomer-generated cascade that activates proteins such as caspase-3, which mediates a non-apoptotic pathway, induces Akt1 inhibition, contributing, in turn, to GSK-3β overactivation [42, 58]. These events are associated with the phosphorylation of threonine-19 of PSD-95, which, in turn, induces the internalization of AMPA receptors from the dendritic spines, facilitating LTD induction [19, 20, 25, 42, 43]. This event could occur independent of plaque formation, preventing neuronal cell death. Such effects are triggered by Aβ oligomers, which are soluble and toxic molecular forms of Aβ [6, 25, 59, 60]; however, these effects might induce GSK-3β inhibition, which might be an independent mechanism (Figure 7). Interestingly, we observed that the presence of ANDRO induce a protection of LTP despite the no change in the basal induction of this process (Figure 5). These evidences indicate that ANDRO modulate the effects of Aβ oligomers over synaptic process inhibiting their action over the synapses, protecting the integrity of the proteins in the synapses, inhibiting the active state of the GSK-3β and promoting the increase in the levels of β-catenin. Therefore, the protection observed *in vitro* is consistent with the observations presented in the animal model of AD, where we found that the presence of ANDRO decrease the active state of GSK-3β and protection of LTP, which is unaltered in WT treatments (Additional file 2: Figure S2). These evidences suggest a regulation in the levels of the active state of GSK-3β, which promote a protection and rescue of the cognitive abilities in a AD model of different ages.

The use of inhibitors or shRNAs that specifically block GSK-3β reduces synapse impairment in AD models [20, 32, 38, 42, 58] similar to the phenomenon observed with the ANDRO treatment. Regarding the possible mechanisms by which ANDRO might exert its effect at the postsynaptic site, we found that ANDRO inhibits LTD in a concentration-dependent manner, preventing the Aβ oligomer mechanism of action (Figure 7). Additionally, GSK-3β inhibition might correlate with the activation of different pathways associated with neuron survival and protection [45]. In this context, *Wnt* signaling is activated after GSK-3β inhibition by lithium, and this phenomenon protects against cognitive impairment [32]. In fact, we demonstrated an increase in the β-catenin levels with ANDRO treatment, suggesting possible activation of the Wnt/β-catenin signaling pathway. Interestingly, some evidence exists that indicates a differential interaction between the NF-κB and the Wnt/β-catenin signaling pathways in an inflammatory context. The mechanism of this interaction proposed GSK-3β as the central regulator between these two pathways. Although GSK-3β inhibits the Wnt/β-catenin pathway by β-catenin degradation and by down-regulation of Wnt target genes, GSK-3β also has the capacity to positively regulate NF-κB by targeting IκB, which inhibits NF-κB, leading to proteasomal degradation [61]. Additionally, β-catenin can interact with the p50 subunit of NF-κB, forming a complex that inactivates the transcriptional activity of NF-κB [62].

In summary, we have identified a natural product that induces a reduction in the active state of GSK-3β, blocking the LTD in the CA1 region of the hippocampus and allowing the recovery of cognitive function in an AD model with different ages (Figure 7). The absence of effective drugs or treatments available for AD motivates the search for new natural products with selective effects on AD models, which might permit an alternative approach to obtaining new therapeutics to treat and to prevent the progression of neurodegenerative diseases such as AD.

## Materials and methods

### Animals

Seven- and twelve-month-old AβPPswe/PS-1 double transgenic male mice expressing mutant AβPPswe (K595N/M596L) and PSEN1ΑE9 transgenes with exon 9 deletion under the control of the mouse prion promoter were obtained from the Jackson Laboratory (Stock #004462). The animals were treated and handled according to the National Institutes of Health guidelines (NIH, Baltimore, MD). Intra-peritoneal (IP) injections of 2.0 mg/kg ANDRO, with saline as the vehicle, were administered three times per week as described in the literature [10, 13, 63]. Transgenic and wild-type (Wt) control animals were injected only with the vehicle. Control Wt data were pooled after analyzing the results because no significant differences were found (data not shown). The experimental procedures were approved by the Bioethical and Biosafety Committee of the Faculty of Biological Sciences of the Pontificia Universidad Católica de Chile, Fondecyt N° 1120156.

### Chemicals and antibodies

Andrographolide (ANDRO) (Additional file 1: Figure S1b) and 6-BIO were obtained from Sigma Chem (St. Louis, MO). The following primary antibodies were used: mouse monoclonal anti-PSD-95 (clone K28/43; (Antibodies, Inc and UC Davis/NIH NeuroMab Facility, Davis, CA), mouse anti-Pan Shank (clone N23B/49; UC Davis/NIH NeuroMab), mouse anti-GluN2B (clone N59/36; UC Davis/NIH NeuroMab), mouse anti-GluA2 (clone L21/32; UC Davis/NIH NeuroMab), goat anti-synaptophysin (sc-7568; Santa Cruz Biotechnology, Inc), rabbit anti-VAMP 1/2/3 (sc-13992; Santa Cruz Biotechnology, Inc), mouse anti-*tau*, paired helical filament (PHF1) epitope (a kind gift from Dr. Peter Davies, Department of Pathology, Albert Einstein College of Medicine, Bronx, NY), mouse anti-β-Actin clone AC-15 (A1978; Sigma-Aldrich), mouse anti-PHF-*tau* clone AT8 (MN1020; Thermo Scientific), rabbit anti-p44/42 MAPK (Erk1/2) (9102; Cell Signaling), rabbit anti-GSK-3β pSer-9 (#9336S; Cell Signaling), anti-GSK-3β pTyr-216 (#612312; BD Biosciences), total GSK-3β (sc-9166; Santa Cruz Biotechnology, Inc), mouse anti-Aβ (4G8) and anti-Aβ oligomer (A11) (Chemicon, Temecula, CA).

### Behavioral tests

#### Classical model test

The Morris water maze (MWM) task was performed as previously described in our laboratory [8, 32, 34, 64, 65]. Briefly, male mice were trained in a 1.2 m diameter circular pool (opaque water, 50 cm deep) filled with water at 19-21°C. A submerged 9-cm platform (1 cm below the surface of water, invisible to the animal) was used for training, with a maximum trial duration of 60 sec and with 10 sec on the platform at the end of the trials. Each animal was trained to locate the platform. The test was performed with three trials per day, and swimming was monitoring using an automatic tracking system (HVS Imagen, Hampton, UK). This system was used to measure the latency time required to reach the platform and the time spent in each quadrant. After testing, the mouse was gently removed from the maze and returned to its cage.

#### Memory flexibility test

The MWM was performed in our laboratory as previously described [32]. Briefly, mice were trained in a 1.2-m diameter circular water maze (opaque water, 50 cm deep, 19–21°C, with a 9-cm platform at 1 cm below water, a maximum trial duration of 60 s, 10 s on the platform at the end of the trials and a delay time between 10 to 15 min). Each animal was trained for one pseudo-random location of the platform per day for 4 days, with a new platform location each day. Up to 10 training trials were performed per day, until the criterion of 3 successive trials with an escape latency of <20 s was met. Upon testing completion, the mouse was removed gently from the maze and returned to its cage. The animals were tested for the next location on the following day. Data were collected using a water maze video tracking system (HVS Imagen).

### Immunohistochemical procedures

Perfusion, fixation and free-floating immuno-cytochemical procedures were performed as previously described [32, 34, 64]. Washes and immune reagent dilutions were performed using 0.01 M PBS with 0.2% Triton X-100 (PBS-T) throughout all of the immunohistochemical experiments, with two PBS-T washes per antibody incubation. Sections were pretreated with 0.5% H_2_O_2_ for 30 min to reduce endogenous peroxidase activity, followed by treatment with 3% bovine serum albumin (BSA) at room temperature for 1 h to avoid non-specific binding. Primary antibodies were incubated overnight at 4°C. Primary antibody detection was performed using a Pierce ABC kit (Thermo Fisher Scientific Inc., Rockford, IL). Staining was developed by incubating for 15 min with 0.6% diaminobenzidine, followed by H_2_O_2_ addition (0.01% final concentration). After immunostaining, all sections were mounted on gelatin-coated slides, air-dried, and dehydrated, and cover slips were placed using Canada balsam (Merck, Darmstadt, Germany).

The specific antibody used for immunohistochemistry was anti-phosphorylated *tau* AT8 (1:1000). The sections were pretreated with 0.3% H_2_O_2_ and then incubated in 3% BSA in PBS. The washes and antibody dilutions were performed using 0.4% Triton X-100 in PBS. Immunohistochemistry was performed using the ABC (avidin biotin-HRP complex) method (Vector Laboratories, Burlingame, CA). Free-floating sections were mounted on gelatin-precoated slides, air-dried, dehydrated in graded ethanol and covered using Canada balsam (Merck, Darmstadt, Germany). Image analysis and PHF-1 neuronal counting were performed as previously described [31].

### Thioflavine-S (Th-S) staining

Th-S staining was developed on gelatin-coated slices as previously described [32, 66]. After dehydration and rehydration in a graded series of ethanol and xylol, slices were incubated in distilled water for 10 min and then were immersed in the Th-S solution (0.1% ThS in 70% ethanol) for 5 min. Then, slices were washed twice in 70% ethanol for 30 s, and cover slips were placed using antifade mounting medium in the dark.

### Image analysis

Stained brain sections were photographed using an Olympus BX51 microscope coupled to a MicroPublisher 3.3 RTV camera (QImaging). The luminescence of the incident light and the time of exposure were calibrated to assign pixel values ranging from 0 to 255 to RGB images (no light to full-light transmission), which were used for all of the preparations. The images were loaded into ImageJv.1.40g software (NIH) for analysis. Selecting areas for measurement was performed by manual threshold adjustment or by direct manual selection of ROIs in heterogeneous stains.

### Immunoblotting

The hippocampi and cortices of treated or control transgenic mice were dissected on ice and immediately processed as previously described [32, 66]. Briefly, hippocampal and cortical tissues were homogenized in RIPA buffer (10 mM Tris-Cl, pH 7.4, EDTA 5 mM, 1% NP-40, 1% sodium deoxycholate, and 1% SDS) supplemented with a protease inhibitor mixture and with phosphatase inhibitors (25 mM NaF, 100 mM Na_3_VO_4_ and 30 μM Na_4_P_2_O_7_) using a Potter homogenizer and then passed sequentially through different caliber syringes. Protein samples were centrifuged at 14,000 rpm at 4°C twice for 15 min. Protein concentrations were determined using a BCA protein assay kit (Pierce). Samples were resolved by SDS-PAGE, followed by immunoblotting on PVDF membranes. Western blot assays were performed as previously described [32, 64].

### Slot blot

Slot blot assays were performed as previously described [32]. Briefly, total protein extracts were centrifuged at 20,000 × *g* for 1 h to eliminate fibrillar aggregates. The protein concentration of the soluble fraction was determined, and 6 μg of protein was spotted on a 0.45-μm^2^ nitrocellulose membrane (Millipore), blocked with 0.4% PBS-T gelatin and incubated with the anti-oligomeric antibody A11 (1/5000) for 2 h at 4°C. Slot blots were revealed using the same methodology used for immunoblotting.

### Slice preparation and electrophysiology

Hippocampal slices were prepared according to previously described standard procedures [39, 67]. Briefly, transverse slices (350 μm) from the dorsal hippocampus were cut under cold artificial cerebrospinal fluid gassed 95% O_2_ and 5%CO_2_ (ACSF, in mM: 124 NaCl, 2.6 NaHCO_3_, 10 D-glucose, 2.69 KCl, 1.25 KH_2_PO_4_, 2.5 CaCl_2_, 1.3 MgSO_4_, and 2.6 NaHPO_4_) using a vibratome (Leica VT 1000S, Germany) and incubated in ACSF for 1 h at room temperature. In all experiments, 10 μM picrotoxin (PTX) was added to suppress inhibitory GABA_**A**_ transmission. Slices were transferred to an experimental chamber (2 ml), superfused (3 ml/min, at 20-22°C) with gassed ACSF and visualized by trans-illumination with a binocular microscope (MSZ-10, Nikon, Melville, NY). To evoke field excitatory postsynaptic potentials (fEPSPs), we stimulated the stratum radiatum within 100–200 μm of the recording site using bipolar concentric electrodes (platinum/iridium, 125 μm OD diameter, FHC Inc., Bowdoin, ME) with pulses generated by a stimulator (Axon 700b, Molecular Devices, Sunnyvale, CA) connected to an isolation unit (Isoflex, AMPI, Jerusalem, Israel). The paired pulse facilitation (PPF) index was calculated using the equation ((R2-R1)/R1), where R1 and R2 were the peak amplitudes of the first and second fEPSPs in an interval inter-pulse of 50 ms, respectively. Basal excitatory synaptic transmission was measured using an input/output curve protocol [31], which consisted of eight stimuli ranging from 200 to 900 μA (the interval between stimuli was 10 s). To generate LTP, we used theta burst stimulation (TBS), which consisted of 5 trains of stimuli with an inter-train interval of 20 s. Each train consisted of 10 bursts at 5 Hz, with each burst composed of 4 pulses at 100 Hz. To generate LTD, we used low-frequency stimulation (LFS), which consisted of 900 paired pulses at 1 Hz. Recordings were filtered at 2.0-3.0 kHz, sampled at 4.0 kHz using an A/D converter, and stored using pClamp 10 software (Molecular Devices). Evoked postsynaptic responses were analyzed off-line using analysis software (pClampfit, Molecular Devices), which allowed events to be visually detected and which computed only those events that exceeded an arbitrary threshold.

### Statistical analysis

Data were expressed as mean ± SEM of the values from the number of experiments as indicated in the corresponding figures. Data were evaluated statistically using Student’s t test with Dunnett’s post hoc test or ANOVA followed by Bonferroni’s post hoc test to determine differences between more than two groups. p < 0.05 was considered as statistically significant. All statistical analyses were performed using Prism software (GraphPad Software Inc.).

## Electronic supplementary material

Additional file 1: Figure S1: Andrographolide (ANDRO) is a diterpene of the labdane family purified from Andrographis paniculata. (a) Picture of *Andrographis paniculata.* (b) Molecular structure of andrographolide (ANDRO). (PDF 109 KB)

Additional file 2: Figure S2: Wild-type animals of different ages treated with ANDRO do not exhibit changes in the LTP. (a) LTP generated by TBS in hippocampal CA1 in wild-type slices from 7-month-old mice treated with ANDRO (black circle) or vehicle solution (white circles). (b) LTP generated by TBS in hippocampal CA1 in wild-type slices from 12-month-old mice treated with ANDRO (black circle) or with vehicle solution (white circles). Inset shows representative recordings. The dots and bars are the mean ± SE from 7 different slices, *p < 0.05. (PDF 89 KB)

Additional file 3: Figure S3: ANDRO recovers synaptic proteins, reduce levels of active of GSK-3β and restore the levels of β-catenin. (a) Immunoblots of total postsynaptic proteins (GluN2B, GluA2 and PSD-95) and presynaptic proteins (SYP and VAMP) extracts from the brain slices treated with vehicle solution (ACSF), Aβ-oligomers (1 μM) or Aβ-oligomers plus ANDRO for 1 hour (white, gray and black bars, respectively). The graph corresponds to the densitometric analysis of each postsynaptic and presynaptic proteins normalized against total ERK and compared with the levels of the same protein in ACSF brain slice treatment. (b) Immunoblots of total β-catenin, GSK-3β, inactive form of GSK-3β (pGSK3β^ser9^) and active form of GSK-3β (pGSK3β^tyr216^) proteins extracts from brain slices treated with vehicle solution (ACSF) or Aβ-oligomers (1 μM) for 1 hour (white and gray bars, respectively). (c) Graph corresponds to the densitometric analysis of each postsynaptic and presynaptic proteins normalized against β-actin and compared with the levels of the same protein in AβPP/PS1 control mice, n ≥ 3. *p < 0.05; **p < 0.01; ***p < 0.001. Immunoblots of total β-catenin, GSK-3β, inactive form of GSK-3β (pGSK3β^ser9^) and active form of GSK-3β (pGSK3β^tyr216^) proteins extracts from brain slices treated with Aβ-oligomers (1 μM) or Aβ-oligomers plus ANDRO for 1 hour (gray and black bars, respectively). Graph corresponds to the densitometric analysis of each postsynaptic and presynaptic proteins normalized against β-actin and compared with the levels of the same protein in AβPP/PS1 control mice, n ≥ 3. *p < 0.05; **p < 0.01; ***p < 0.001. (PDF 240 KB)

## Abbreviations

AβPP: Amyloid precursor protein
PS-1: Presenilin-1
Aβ: Amyloid β-peptide
ANDRO: Andrographolide
PSD-95: Postsynaptic density protein-95
SYP: Synaptophysin
GFAP: Glial fibrillary acidic protein
GSK-3β: Glycogen synthase kinase-3β
MWM: Morris water maze
LTP: Long-term potentiation
LTD: Long-term depression
CNS: Central nervous system
BSA: Bovine serum albumin
ACSF: Artificial cerebrospinal fluid
fEPSP: Field excitatory post-synaptic potential
TBS: theta burst stimulation
LFS: Low-frequency stimulation
PHF: Paired helical filaments
PPF: Paired pulse facilitation.
