# Supplementary figures and images for: Andrographolide reduces cognitive impairment in young and mature AβPPswe/PS-1 mice
Source: Mol Neurodegener. 2014 Dec 18;9:61. doi: 10.1186/1750-1326-9-61 (PMC4414355; doi:10.1186/1750-1326-9-61)

a

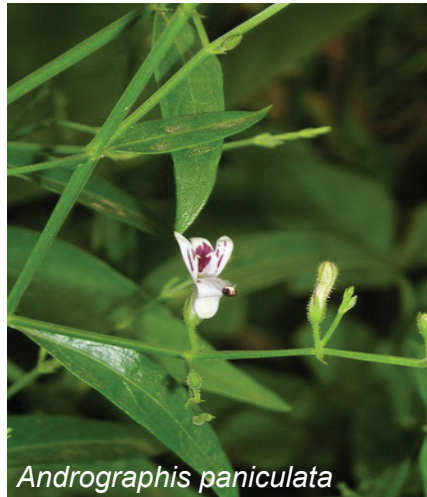

b

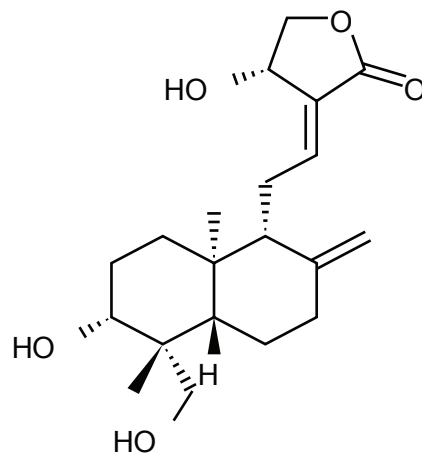

Supplement: Supplementary file 1 — Additional file 1: Figure S1: Andrographolide (ANDRO) is a diterpene of the labdane family purified from Andrographis paniculata. (a) Picture of Andrographis paniculata. (b) Molecular structure of andrographolide (ANDRO). (PDF 109 KB) [file 13024_2014_578_MOESM1_ESM.pdf]

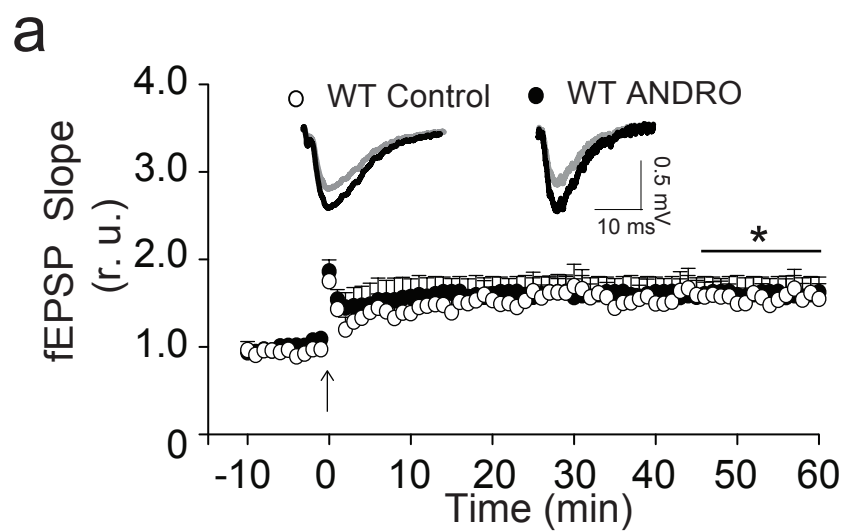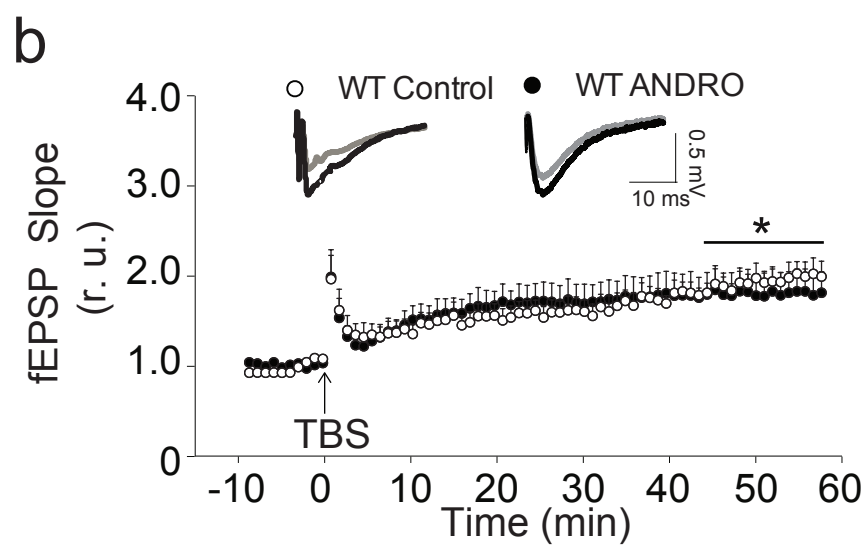

Supplement: Supplementary file 2 — Additional file 2: Figure S2: Wild-type animals of different ages treated with ANDRO do not exhibit changes in the LTP. (a) LTP generated by TBS in hippocampal CA1 in wild-type slices from 7-month-old mice treated with ANDRO (black circle) or vehicle solution (white circles). (b) LTP generated by TBS in hippocampal CA1 in wild-type slices from 12-month-old mice treated with ANDRO (black circle) or with vehicle solution (white circles). Inset shows representative recordings. The dots and bars are the mean ± SE from 7 different slices, *p < 0.05. (PDF 89 KB) [file 13024_2014_578_MOESM2_ESM.pdf]

**a**

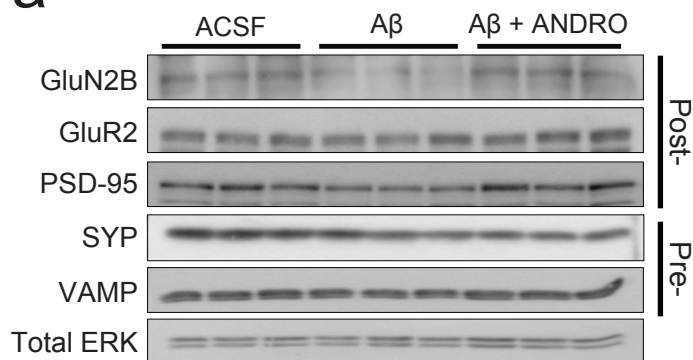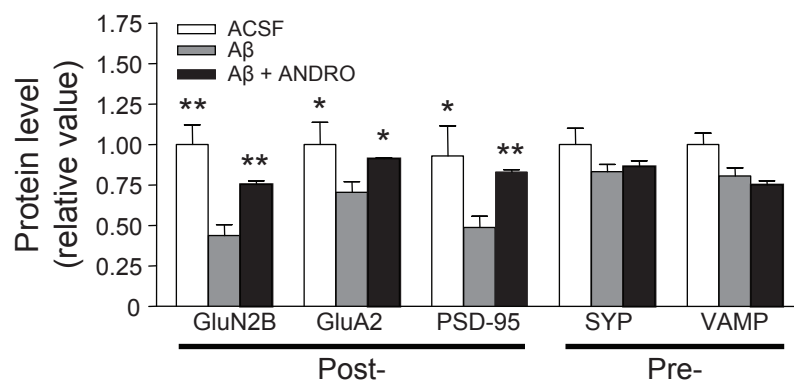

**b**

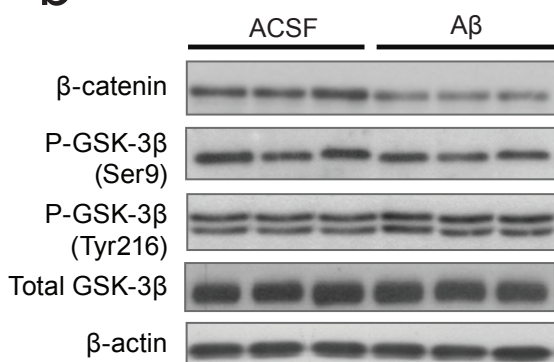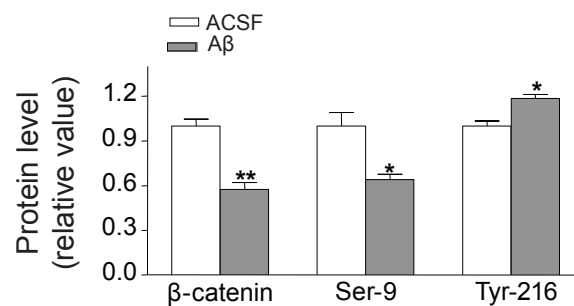

**c**

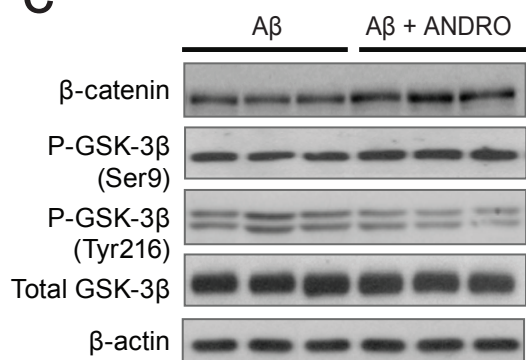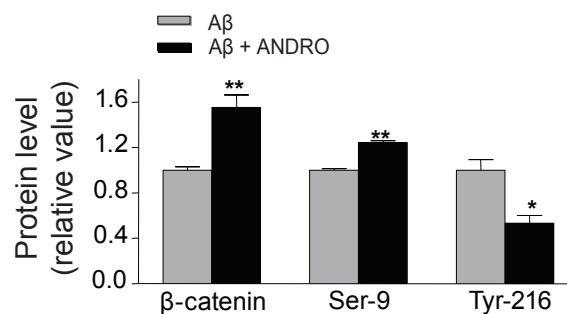

Supplement: Supplementary file 3 — Additional file 3: Figure S3: ANDRO recovers synaptic proteins, reduce levels of active of GSK-3β and restore the levels of β-catenin. (a) Immunoblots of total postsynaptic proteins (GluN2B, GluA2 and PSD-95) and presynaptic proteins (SYP and VAMP) extracts from the brain slices treated with vehicle solution (ACSF), Aβ-oligomers (1 μM) or Aβ-oligomers plus ANDRO for 1 hour (white, gray and black bars, respectively). The graph corresponds to the densitometric analysis of each postsynaptic and presynaptic proteins normalized against total ERK and compared with the levels of the same protein in ACSF brain slice treatment. (b) Immunoblots of total β-catenin, GSK-3β, inactive form of GSK-3β (pGSK3βser9) and active form of GSK-3β (pGSK3βtyr216) proteins extracts from brain slices treated with vehicle solution (ACSF) or Aβ-oligomers (1 μM) for 1 hour (white and gray bars, respectively). (c) Graph corresponds to the densitometric analysis of each postsynaptic and presynaptic proteins normalized against β-actin and compared with the levels of the same protein in AβPP/PS1 control mice, n ≥ 3. *p < 0.05; **p < 0.01; ***p < 0.001. Immunoblots of total β-catenin, GSK-3β, inactive form of GSK-3β (pGSK3βser9) and active form of GSK-3β (pGSK3βtyr216) proteins extracts from brain slices treated with Aβ-oligomers (1 μM) or Aβ-oligomers plus ANDRO for 1 hour (gray and black bars, respectively). Graph corresponds to the densitometric analysis of each postsynaptic and presynaptic proteins normalized against β-actin and compared with the levels of the same protein in AβPP/PS1 control mice, n ≥ 3. *p < 0.05; **p < 0.01; ***p < 0.001. (PDF 240 KB) [file 13024_2014_578_MOESM3_ESM.pdf]
